# Will an Implementation of “Joy of Life in Nursing Homes” Have Positive Effect for the Work Culture? A Comparison Between Two Norwegian Municipalities

**DOI:** 10.3389/fpsyg.2020.607943

**Published:** 2021-02-04

**Authors:** Beate André, Frode Heldal, Endre Sjøvold, Gørill Haugan

**Affiliations:** ^1^Department of Public Health and Nursing, Norwegian University of Science and Technology, Trondheim, Norway; ^2^NTNU Center for Health Promotion Research, Trondheim, Norway; ^3^NTNU Business School, Trondheim, Norway; ^4^Department of Industrial Economics and Technology Management, Norwegian University of Science and Technology, Trondheim, Norway; ^5^Faculty of Nursing and Health Sciences, Nord University, Levanger, Norway

**Keywords:** implementation, nursing homes, work culture, quality of care, job satisfaction

## Abstract

**Background:**

Currently, we are facing a demographic shift to an older population and its consequences worldwide: in the years to come, several older people will need nursing home (NH) care. The work culture is important for care quality in NHs. Some Norwegian municipalities have implemented the Joy of Life Nursing Home (JoLNH) strategy, representing a resource-oriented health-promoting approach. Knowledge about how implementation of the JoLNH approach impacts the work culture is scarce.

**Aimss:**

We hypothesized that the JoLNH strategy impacts positively on the work culture: (1) when comparing measurements at two time points (T1 and T2) and (2) when comparing two municipalities, among which one has implemented the JoLNH and the other has not.

**Method:**

With a 1-year interval, healthcare personnel in 43 NHs located in two large Norwegian municipalities responded to a survey in two waves (T1 and T2). In total, 558 healthcare personnel participated at T1 and 515 at T2. Work culture was assessed by the Systematizing Person–Group Relations Instrument (SPGR).

**Findings:**

The municipality implementing the JOLNH strategy experienced significant increases in SPGR dimensions, representing positive orientations towards a better work culture, whereas those working in the non-JoLNH municipality reported an increase in SPGR dimensions, signifying a worsened work culture.

**Discussion:**

The data indicate that the implemented JoLNH strategy has endorsed positive effects in the work culture. The JoLNH strategy emphasizes on the quality of patient care, which is what healthcare personnel, in general, are much concerned about. Furthermore, attention to task orientation and independent work seem to encourage a better work culture in NHs.

**Conclusion:**

This study suggests that the JoLNH strategy impacts positively on the work culture in Norwegian NHs.

## Background

Currently, we are facing a demographic shift to an older population and its consequences worldwide: in the years to come, several older people will need nursing home (NH) care. The work culture in NHs affects healthcare workers and the care quality. Therefore, focusing on the NH work culture is vital (WHO, 2018).

### Work Culture

Work culture is defined as the norms, values, and basic supposition joint by associates of a group (Gershon et al., 2004; Sjøvold, 2007), whereas work climate states to the awareness of administrative challenges such as decision-making, leadership, and norms concerning the work (Gershon et al., 2004; Stone et al., 2005; Andre et al., 2012; André et al., 2013b; Andre et al., 2016; André and Sjøvold, 2017). As used in this article, the concept of “work culture” covers both culture and climate.

### The Implementation of the JoLNH Strategy

In 2019, 40,000 people were living in Norwegian NHs (Statistics Norway SSB, 2020); in the years to come, this number is expected to increase. To meet the increasing need of NH care and simultaneously provide care quality, the national strategy Joy of Life Nursing Home (JoLNH) was established in Norway. The JoLNH is founded on a health-promoting perspective focusing on the older persons’ resources (Rinnan et al., 2018): NH residents’ well-being, meaning-in-life, and quality of life represent the main aims (Helse- og omsorgsdepartementet, 2012–2013). As recommended by the Norwegian government (Helse- og omsorgsdepartementet, 2012–2013), several municipalities have implemented this strategy. To become an approved JoLNH, nine claims must be realized (Rinnan et al., 2018): (1) all staff must have knowledge about the JoLNH philosophy and its implications, (2) the NH has an obligation to collaborate with schools, kindergartens, and other organizations, (3) arrange for outdoors enjoying fresh air at a minimum of once a week to all residents, (4) enable interaction with animals if wanted, (5) enable the residents to uphold their hobbies and interests if desired, (6) facilitate musical and cultural enjoyment and (7) a nice atmosphere throughout mealtimes, (8) respectable communication with family and next of kin, and, finally, (9) the NH must facilitate that seasons and holidays are visible in the regular routines (Livsglede for eldre, 2016). The Joy-of-Life (JoL) foundation (Livsglede for eldre, 2016) conducts the JoLNH implementation, including the certification process, together with the NHs and the municipality. When implementing the JoLNH strategy, the municipality and the individual NHs must make the NH ready for the certification process by fulfilling three requirements (Livsglede for eldre, 2016; Rinnan et al., 2018; Haugan et al., 2019, 2020):

1. The municipalities must pay a fee to the JoL foundation for the certification as well as the recertification processes.
2. The municipality employs two to three JoL-coordinators who assist the NHs in the certification and recertification processes.
3. The NH must work through a recertification process annually.

The present study was conducted in collaboration with two large municipalities, among which one was implementing the JoLNH (experimental municipality) while the other did not (control municipality). During the study period, the NHs in the experimental municipality were at different stages; that is, some were newly certified JoLNHs, some were recertified, and some were in the first year of implementation. The researchers were independent of those who implemented the JoLNH and the municipality.

### Changes and Implementation

Taking part in changes and implementations influencing one’s working routines and approaches may be challenging but can also be a positive event (Andre et al., 2016). Structural willingness for change is considered a criterion for successful implementations in healthcare (Weiner, 2009; Schultz et al., 2017a). The concept of structural willingness for change can be described as attitudes, intents, values, principles, and behavior among people in an organization which are related to how the management is trying to influence on the change process (Weiner, 2009; Jacobs et al., 2015; Richards and Hallberg, 2015; Schultz et al., 2016, 2017b). Implementation and changes might be time-consuming and lead to stress for healthcare personnel (André et al., 2016). Both performance and purposes are well labeled and prejudiced by numerous issues such as attitudes, standards, and enthusiasm (Strobe, 2008).

### Objectives

Research highlights the importance of facilitating the implementation process for healthcare personnel (André et al., 2008a,b, 2017; Andre et al., 2009; Damschroder et al., 2009; André, 2012; Lewy, 2015; André and Sjøvold, 2017; Schultz et al., 2017a). To achieve a successful implementation, the work culture during the implementation is crucial. Therefore, this study investigates the work culture in NHs by testing the following hypotheses:

1. Hypothesis 1 (H1): The JoLNH strategy impacts positively on the work culture in the experimental municipality, comparing scores at two different time points (T1 and T2).
2. Hypothesis 2 (H2): The JoLNH strategy impacts positively on the work culture when comparing the experimental municipality with the control municipality.

## Materials and Methods

The present study is a pilot aimed at observing trends to be applied under conditions of greater control.

### Subjects and Data Collection

Out of 2,835 (T1) and 3,221 (T2) invited participants, 558 (T1) and 515 (T2) healthcare personnel in the 43 NHs located in two large central municipalities responded twice (T1 = winter 2015/2016 and T2 = winter 2016/2017) to the questionnaire involving demographic data and the Systematizing Person–Group Relations Instrument (SPGR). The response rates were 20% and 16%, respectively. The two data collections (T1 and T2) were conducted similarly at the same time period each year (Grødal et al., 2019a).

The respondents received a link to the questionnaire by e-mail, responded, and returned the questionnaire at the website (Grødal et al., 2019a,b). The same healthcare personnel received the invitation to participate at T1 and T2. However, during a 1-year period, ordinary movements such as retirements, layoffs, abandonment, and entry of new workers will occur, which may explain why the number of invitations is higher at T2 than at T1. Table 2 provides a more detailed description of the subjects.

### Measurements and Data Analysis

The SPGR instrument was developed in Norway by Sjøvold (2004, 2006, 2007) assessing work culture. The respondents were asked to describe the work culture today by rating the specific behavior items. In this study, we conducted a two-tailed Student’s *t*-test for significance between the two independent tests (Sjøvold, 2007). The two datasets were approximately normally distributed. The SPGR instrument has shown acceptable values for reliability (Cronbach’s alpha, 75–86) and validity, particularly predictive validity (Sjøvold, 2007).

**FIGURE 1:**
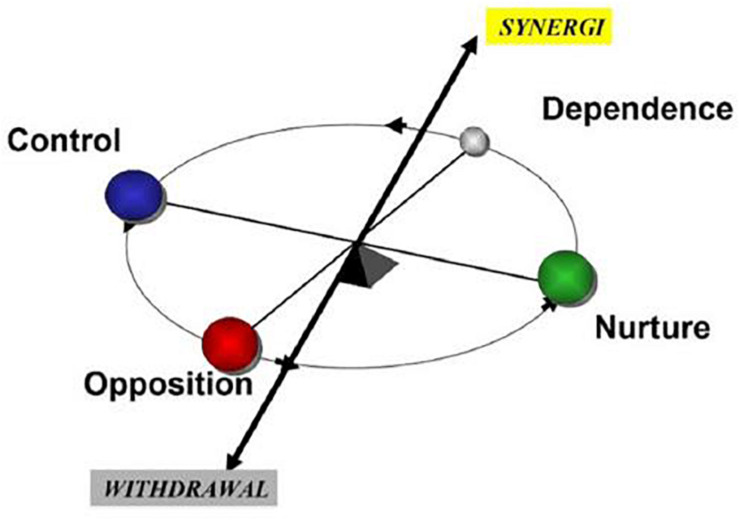
The SPGR instrument relies on a factor analytical model consisting of these basic dimensions.

Each item is rated according to whether the specific behavior occurred (1) never or seldom, (2) sometimes, and (3) often or always. The numbers describe a mean value on a linear scale from 1 to 9. In SPGR, the organizational culture is described by the organization’s predominant behaviors. The organizational behaviors are described along dimensions labeled as follows: control and nurture (C–N), opposition and dependence (O–D), and withdrawal and synergy (W–S); each dimension has two vectors (Sjøvold, 2007) as shown in Table 1 and Figure 1.

**TABLE 1:** Elements of group constitution based on the Systematizing Person–Group Relations instrument.

Dimension/vectors		Group function	Short description
C–N	C	Control	Structure, logic, authority
	N	Nurture	Caring, social orientation, openness
O–D	O	Opposition	Criticism, rebellion
	D	Dependence	Loyalty, conformance, submission
W–S	W	Withdrawal	Passive resistance
	S	Synergy	Engagement, constructive goal-oriented

**TABLE 2:** Demographic data in the experimental municipality and the control municipality (given in %) in two different waves (T1 and T2).

	JoLNH (1)		Not JoLNH (2)	
Year	2016 T1	2017 T2	2016 T1	2017 T2
**Gender**				
Female	90	90	88	88
Male	10	10	12	12
**Education**				
Less than 3 years in a university	48	47	32	32
Three years in a university	33	34	34	34
More than 3 years in a university	19	19	34	34
**Age**				
20–39 years	45	43	27	30
40–59 years	39	40	49	46
Over 60 years	16	17	24	24
**Job title**				
Manager	11	11	28	28
Nurse	35	35	44	44
Assistant nurses	46	46	40	40
Assistant	13	13	14	14
Other	5	5	2	2
**Speaking native Norwegian**				
Yes	90	90	88	89
No	10	10	12	11

The SPGR reveals characteristics of the work culture in the organizational units: The *control* dimension is described by analytical, task-oriented, or autocratic behavior. The *nurture* dimension is in focus when caring, empathic, or spontaneous behavior dominates. The *opposition* dimension is characterized by critical, assertive, or self-sufficient behavior. Passive and obedient behavior is in focus in the *dependence* dimension, and when engagement and constructive goal orientation behavior dominates, *synergy* is highlighted. The *withdrawal* dimension is characterized by restriction from contribution and commitment to a initial role involving dominant behavior (Sjøvold, 2006; Andre et al., 2012;, André et al.2016, 2017).

The SPGR is a “balance model,” implying that when one specific kind of behavior, e.g., loyalty and acceptance, is much used, then the opposite behavior (criticism and assertiveness) is scarce (Sjøvold, 2004; Andre et al., 2012, 2013a). Figure 1 portrays the SPGR “balance model.”

### Ethical Considerations

All participants received information about the aim and purpose of the study and consented to participate by responding to the questionnaire voluntarily and anonymously. The Norwegian Centre for Research Data, Data Protection Services, was notified of the project. In accordance with the Norwegian Health Research Act, approval by the Regional Committee for Medical and Health Research Ethics was not required.

## Results

The two Norwegian municipalities included were of approximately similar size and population. Table 2 shows a list of the demographics of the two municipalities.

The demographic data revealed minor changes from T1 (year 2016) to T2 (2017) inside each municipality as well as no big differences between the two municipalities. Nonetheless, the healthcare workers in the municipality without an implementation of JoLNH (control municipality) were older than those in the experimental municipality (implementing the JoLNH) and comprised more nurses and managers, fewer assistant nurses, and other professional groups. Differences between T1 and T2 of perceived work culture in the JoLNH municipality are shown in Table 3.

**TABLE 3:** Differences in perceived work culture in the experimental municipality.

Vector	Code	Typical behavior	T1	T2
Ruling	C1	Controlling, autocratic, attentive to rules and procedures	3.71	3.76
Task-orientation	C2	Analytical, task-oriented, conforming	4.75	4.94^a^
Caring	N1	Taking care of others, attentive to relations	5.20	5.32
Creativity	N2	Creative, spontaneous	2.79	2.91
Criticism	O1	Critical, opposing	3.00	3.04
Assertiveness	O2	Assertive, self-sufficient	3.08	3.06
Loyalty	D1	Obedient, conforming	5.13	5.30^a^
Acceptance	D2	Passive, accepting	5.19	5.33
Resignation	W1	Sad appearance, showing lack of self-confidence	2.82	2.79
Self-sacrifice	W2	Passive, reluctant to contribute	2.65	2.70
Engagement	S1	Engaged, inviting others to contribute	5.29	5.38
Empathy	S2	Showing empathy and interest in others	5.25	5.39^a^

*^*a*^Statistically significant difference at *p* < 0.05.*

### The Experimental Municipality: Differences in Work Culture

D1 (obedient, conforming behavior), along with S2 (empathy and interest in others) and C2 (task-oriented, conforming behavior), disclosed significantly higher values at T2 than at T1. Accordingly, 1 year after, the healthcare personnel were more loyal to tasks, willingly working, following norms, trustworthy, and precise (D1) as well as more creative, intuitive, dramatic, spontaneous, looking for absurdity, experimenting, and breaking norms (N2) (although the latter value is not significant). The first one (D1) could be an indicator of more compliant behavior, while the latter one (N2) could be an indicator of searching for new solutions. The significant increase in task orientation (C2), together with a significant increase in caretaking and empathy (S2), may be interpreted as a sign that the work culture has at least some positive developments, which affect the healthcare workers’ perceived ability to perform. Although findings must be more contextualized, this may be a matter of going along with the process, showing loyalty to the implementation process (D2) – and showing positive feelings with regards to this change.

### The Control Municipality: Differences in Work Culture

Table 4 shows a comparison between T1 and T2 for the control municipality. Similar to the experimental municipality, creativity (N2) has increased. However, D1 shows a decrease. Compared to T1, the respondents are thus less compliant to follow the decisions; probably they are turning to their own solutions. Table 3 presents notable results, exposing an increase of W1 (sad appearance) and W2 (passive, reluctant to contribute) – signifying resignation and withdrawing from collaboration. W1 and W2 are indications of behaviors that show non-talkative, not participating, non-responsive, passive, low confidence (W1) and sad, complaining, sorrow and disbelief, and self-pitying (W2).

**TABLE 4:** Differences in perceived work culture in a municipality (2) without implementation of JOLNH.

Vector	Code	Typical behavior	T1	T2
Ruling	C1	Controlling, autocratic, attentive to rules and procedures	3.67	2.95^b^
Task-orientation	C2	Analytical, task-oriented, conforming	4.88	2.79
Caring	N1	Taking care of others, attentive to relations	5.35	2.51^b^
Creativity	N2	Creative, spontaneous	2.70	2.42^a^
Criticism	O1	Critical, opposing	2.86	5.21^b^
Assertiveness	O2	Assertive, self-sufficient	2.99	2.57^b^
Loyalty	D1	Obedient, conforming	5.22	5.41
Acceptance	D2	Passive, accepting	5.27	2.69^b^
Resignation	W1	Sad appearance, showing lack of self-confidence	2.66	5.37^b^
Self-sacrifice	W2	Passive, reluctant to contribute	2.49	5.29^b^
Engagement	S1	Engaged, inviting others to contribute	5.47	3.58^b^
Empathy	S2	Showing empathy and interest in others	5.38	4.79^b^

*^*a*^Statistically significant difference at *p* < 0.05. ^*b*^Statistically significant difference at *p* < 0.01.*

The relatively high decrease in C1 (analytic, structured, logical, task-oriented, time-oriented, neutral, and punctual behaviors), along with the above-mentioned findings (W1 and W2), suggests that the individuals are orienting themselves away from the organization and system.

Furthermore, the control municipality shows significant differences in all dimensions except D1 – which may be attributed to behavior that is loyal to the managers and thus following the norms. Although not significant, they even show an increase. This can be an indication about workers doing their best to perform their tasks and duties despite a culture that is challenging. A more challenging work culture is depicted by all dimensions (except D1). The combined C dimensions may represent behaviors that are oriented towards performance, going by the book, and following authorities. Both are decreasing, which may be a sign of wanting deliberation or emancipation from what others have decided (most often authoritative leaders). The combined N dimensions may represent behaviors that are easily linked to perceived culture – that is, care, fun, and feeling good at work. Both are significantly decreasing. The increase in the O1 vector may signal that people start to openly criticize either each other, leaders, or other things. Together with a decrease in the O2, which denotes the ability to speak up and state individual desires, this may suggest that people are feeling suppressed. Nevertheless, as previously stated, despite feeling suppressed, the healthcare professionals are following the work orders; probably, they feel obliged to provide the best care quality as possible. The significant increase in both W vectors is troublesome, indicating that people are withdrawing from their work, calling in sick, and/or being sick. The more positive S dimensions are both showing significant decreases. All in all, these findings attest to a significant worsened work culture.

### Differences in Work Culture Between the Two Municipalities at T2

Table 5 discloses significant differences between the municipalities at T2. Dimensions that can be contributed to a heightened or improved work culture reveal significantly higher scores, accompanied by significantly lesser behaviors attributed to negative feelings (W1 and W2) and significantly more behaviors attributed to a constructive and positive culture (S1 and S2). It appears that the individuals experience a significantly better work culture at T2, followed by caretaking (N1 and N2) and a structured attention to getting the work done (C1 and C2).

**TABLE 5:** Comparison between two municipalities, with implementation (exploratory) and without implementation (control) at T2.

Vector	Code	Typical behavior	T2 (1)	T2 (2)
Ruling	C1	Controlling, autocratic, attentive to rules and procedures	3.76	2.95^a^
Task-orientation	C2	Analytical, task-oriented, conforming	4.94	2.79^a^
Caring	N1	Taking care of others, attentive to relations	5.32	2.51^a^
Creativity	N2	Creative, spontaneous	2.91	2.42^a^
Criticism	O1	Critical, opposing	3.04	5.21^a^
Assertiveness	O2	Assertive, self-sufficient	3.06	2.57^a^
Loyalty	D1	Obedient, conforming	5.30	5.41
Acceptance	D2	Passive, accepting	5.33	2.69^a^
Resignation	W1	Sad appearance, showing lack of self-confidence	2.79	5.37^a^
Self-sacrifice	W2	Passive, reluctant to contribute	2.70	5.29^a^
Engagement	S1	Engaged, inviting others to contribute	5.38	3.58^a^
Empathy	S2	Showing empathy and interest in others	5.39	4.79^a^

*^*a*^Statistically significant difference at *p* < 0.05.*

## Discussion

This study focused on the possible influences of implementing the JoLNH strategy. We investigated two hypotheses: H1: the JoLNH strategy impacts positively on the experimental municipality, comparing the measurements at two time points (one year interval), and H2: the JoLNH strategy impacts positively on the work culture when comparing the experimental municipality with the control municipality (not applying the JoLNH strategy).

The demographic data show minor changes in the employment group in both municipalities with and without implementation of JoLNH. The demographic differences between the municipalities relate to age and the different professions employed. However, these differences may not explain the significant differences found in the work culture.

The JoLNH strategy impacts positively on the experimental municipality, comparing the measurements at two time points (H1).

Our findings revealed a largely stable work culture in the exploratory municipality. However, from T1 to T2, some changes appeared: if C2 and S2 do not create an imbalance in relation to the other vectors, task orientation (C2) and empathy (S2) are considered as positive qualities in a work culture. Empathy and engagement belong to the “synergy” dimension, which is important for achieving high maturity in both the independent work and collaboration aspects (Sjøvold, 2006). An increase in these dimensions was displayed, signifying that the work culture in the participating units has developed positively during the study period (T1–T2). During an implementation process, D1 (loyalty) is important; the present study revealed that D1 increased. Our findings imply that the JoLNH strategy impacts positively on the work culture in the experimental municipality, comparing the measurements at two different time points (H1).

The JoLNH strategy impacts positively on the work culture when comparing the experimental municipality with the control municipality (H2).

Differences between the two municipalities in eight of the 12 dimensions were found at T2. The experimental municipality (implementing JoLNH) demonstrated a stable work culture with nearly similar findings at T1 and T2, except for an increase in C2 (analytical, task-oriented, conforming) and S2 (showing empathy and interest in others). Along with the demographical findings showing few differences between T1 and T2 for the experimental municipality, these findings indicate a stable work culture.

The control municipality (not implementing JoLNH) exposed significant differences at T1 and T2, with a decrease in the dimensions representing positive values in the work culture (C2, N1, S1, and S2) and an increase in the dimensions embodying negative aspects of the work culture (O1, D1, W1, and W2) (Table 5).

Despite the fact that the healthcare personnel in the experimental municipality were not offered possibilities to influence on the decision of implementing the JoLNH, it seems that the JoLNH implementation has involved values of influence and participation in decisions. Moreover, the JoLNH’s emphasis on individually personalized care may initiate a greater influence on nursing interventions among healthcare professionals (Rinnan et al., 2018; Haugan et al., 2020). Furthermore, the healthcare personnel’s experience of providing good care quality may result from closer relationships with the NH patients (André and Jacobsen, 2020).

In healthcare settings, implementation and changes may be stressful for healthcare workers (Andre et al., 2009; Weiner, 2009; André, 2012; Jacobs et al., 2015; Richards and Hallberg, 2015; André et al., 2016; André and Sjøvold, 2017). However, this this seems not to be the case in this JoLNH implementation since the work culture in the experimental municipality is slightly improved (Tables 3, 5). This improvement can result from both the facilitation of the implementation process and/or the content and quality of the JoLNH strategy. The healthcare personnel may consider the JoLNH strategy as an opportunity to deliver more high-quality patient care. The JoLNH strategy strongly focuses on personalized interventions for individual NH residents: hence, strengthened empowerment and influence on decisions may also result from implementing the JoLNH (Rinnan et al., 2018; Haugan et al., 2020). This possibly explains the increase in C2 (Table 3), revealing a stronger emphasis on analytical, task-oriented, and conforming behavior. These aspects are important in developing a sound work culture (Uğur et al., 2007; Nøst et al., 2016). The present findings support that the JoLNH strategy impacts positively on work culture when comparing the experimental municipality with the control municipality (not implementing JoLNH) (H2).

### Methodological Discussion

Interpretation of replication studies requires an assumption of psychometric invariance across the original and replication experiments. If psychometric invariance is severely violated, one cannot expect a replication experiment to reproduce the findings of the original experiment (Fabrigar and Wegener, 2016). A replication of this study will lead to findings other than those presented here: both the work culture and healthcare personnel’s experiences of work culture will be subject to influence by factors such as retirement, layoffs, abandonment, entry of new workers, workload, and change in work descriptions. However, when introducing JoLNH in some units or municipalities and comparing with others without this implementation, there is a possibility that our findings may be replicated because of the differences that we have found between the two municipalities in this study.

### Limitations

This study only considered the implementation of JoLNH related to the work culture. However, different municipalities have implemented different strategies in their NHs; these strategies were not considered in this study. Thus, it is plausible that other factors which we did not assess have influenced on the present results. The response rates were low, which is a limitation of this study. However, since the mailing lists were not updated, not all healthcare personnel might have received the questionnaire. Moreover, having various engagements, the healthcare personnel might have received several requests and thus missed out to respond to our survey. Consequently, the response rates were probably higher. Hence, the sampling process may have influenced the results if only the most enthusiastic healthcare personnel participated in one municipality and the opposite in the other. The researchers were independent of both those who implemented JoLNH and the municipality. Therefore, it is not possible to state if the implementation process was similar in every single NH. This study was conducted in Norway with a Norwegian population. The work conditions in Norway are usually favorable for workers; hence, generalizing the present results must be considered carefully.

## Conclusion

This study describes the differences in the work culture of a municipality that has implemented the JoLNH strategy and a municipality that has not implemented this strategy. Implementations and changes are reasons of concern by the management of the municipalities and have been looked upon as a challenge to the work culture. This study suggests that the JoLNH implementation did not lead to any negative outcomes for the work culture in the NHs participating in this study (H1). Differences between the municipalities were found, and this study supported that the JoLNH strategy will have a positive impact when comparing the experimental municipality with the control municipality which did not implement the JoLNH (H2).

## Data Availability Statement

The original contributions presented in the study are included in the article/supplementary material, further inquiries can be directed to the corresponding author.

## Ethics Statement

Participation in this study was voluntary for the respondents, and they were informed about the aim and purpose of the study. All registration of the participants was anonymous, and respondents gave their consent by responding to the questionnaire. The Norwegian Centre for Research Data, Data Protection Services, was notified of the project. Prior to this, an application was sent to the Regional Committee for Medical and Health Research Ethics, who declared that approval for the current project was not required according to the Norwegian Health Research Act.

## Author Contributions

BA, GH, and ES conceived the study, designed the survey, and supervised the work. BA and GH collected the data. ES and FH analyzed the data. FH and ES performed all statistical analyses. BA and FH prepared the initial draft of the manuscript. FH, ES, and BA created figures and tables. All authors interpreted the data, critically revised the manuscript, and gave final approval for publication.

## Conflict of Interest

The authors declare that the research was conducted in the absence of any commercial or financial relationships that could be construed as a potential conflict of interest.

## References

[B1] André B. (2012). “Challenges among health care workers when changes are introduced,” in *Health Promotion – Theory and Practice*, Vol. 1 ed. Innstrand S. T. (Trondheim: Reseach Centre for health Promotion and Resourses HiST/NTNU), 37–47.

[B2] André B. Frigstad S. A. Nøst T. H. Sjøvold E. (2016). Exploring nursing staffs communication in stressful and non-stressful situations. *J. Nurs. Manag.* 24 E175–E182. 26077500 10.1111/jonm.12319

[B3] André B. Jacobsen F. F. (2020). The art of caring in selected norwegian nursing homes: a qualitative approach. *Int. J. Caring Sci.* 13 820–827.

[B4] André B. Nøst T. H. Frigstad S. A. Sjøvold E. (2017). Differences in communication within the nursing group and with members of other professions at a hospital unit. *J. Clin. Nurs.* 26 956–963. 27240269 10.1111/jocn.13410

[B5] André B. Ringdal G. I. Loge J. H. Rannestad T. Kaasa S. (2008a). The importance of key personnel and active management for successful implementation of computer-based technology in palliative care: results from a qualitative study. *Comput. Inform. Nurs.* 26 183–189. 10.1097/01.NCN.0000304802.00628.70 18600124

[B6] André B. Ringdal G. I. Loge J. Rannestad T. Laerum H. Kaasa S. (2008b). Experiences with implementation of computerized tools in health care units – a review article. *Int. J. Hum. Comput. Interact.* 24 753–775. 10.1080/10447310802205768

[B7] Andre B. Ringdal G. I. Skjong R. J. Rannestad T. Sjøvold E. (2016). Exploring experiences of fostering positive work environment in Norwegian nursing homes: a multi method study. *Clin. Nurs. Stud.* 4:9.

[B8] Andre B. Ringdal G. I. Loge J. H. Rannestad T. Kaasa S. (2009). Implementation of computerized technology in a palliative care unit. *Palliat Support Care* 7 57–63. 10.1017/S147895150900008X 19619375

[B9] André B. Sjøvold E. (2017). What characterizes the work culture at a hospital unit that successfully implements change–a correlation study. *BMC Health Serv. Res.* 17:486. 10.1186/s12913-017-2436-4 28705155 PMC5513374

[B10] Andre B. Sjøvold E. Holmemo M. Rannestad T. Ringdal G. I. (2013a). Expectations and desires of palliative health care personnel concerning their future work culture. *J. Hosp. Adm.* 2:46 10.5430/jha.v2n3p46

[B11] Andre B. Sjovold E. Rannestad T. Holmemo M. Ringdal G. I. (2012). Work culture among healthcare personnel in a palliative medicine unit. *Palliat Support Care* 11 135–140. 10.1017/S1478951512000818 23089522

[B12] André B. Sjøvold E. Rannestad T. Ringdal G. I. (2013b). The impact of work culture on quality of care in nursing homes – a review study. *Scand. J. Caring Sci.* 28 449–457. 10.1111/scs.12086 24117657

[B13] Damschroder L. J. Aron D. C. Keith R. E. Kirsh S. R. Alexander J. A. Lowery J. C. (2009). Fostering implementation of health services research findings into practice: a consolidated framework for advancing implementation science. *Implement. Sci.* 4:50. 10.1186/1748-5908-4-50 19664226 PMC2736161

[B14] Fabrigar L. R. Wegener D. T. (2016). Conceptualizing and evaluating the replication of research results. *J. Exp. Soc. Psychol.* 66 68–80. 10.1016/j.jesp.2015.07.009

[B15] Gershon R. R. M. Stone P. W. Bakken S. Larson E. (2004). Measurement of organizational culture and climate in healthcare. *J. Nurs. Admin.* 34 33–40. 10.1097/00005110-200401000-00008 14737033

[B16] Grødal K. Innstrand S. T. Haugan G. André B. (2019a). Affective organizational commitment among nursing home employees: a longitudinal study on the influence of a health-promoting work environment. *J. Nurs. Open* 6 1414–1423. 10.1002/nop2.338 PMC6805324 31660169

[B17] Grødal K. Innstrand S. T. Haugan G. André B. (2019b). Work-related sense of coherence and longitudinal relationships with work engagement and job satisfaction. *Scand. J. Work Organ. Psychol.* 4:5.

[B18] Haugan G. Eide W. M. André B. Wu V. X. Rinnan E. Taasen S. E. (2020). Joy-of-life in cognitively intact nursing home patients: the impact of the nurse–patient interaction. *Scand. J. Caring Sci.* 10.1111/scs.12836 32200564

[B19] Haugan G. Rinnan E. Espnes G. A. Drageset J. Rannestad T. André B. (2019). Development and psychometric properties of the Joy-of-Life Scale in cognitively intact nursing home patients. *Scand. J. Caring Sci.* 33 801–814. 30866075 10.1111/scs.12676

[B20] Jacobs S. R. Weiner B. J. Reeve B. B. Hofmann D. A. Christian M. Weinberger M. (2015). Determining the predictors of innovation implementation in healthcare: a quantitative analysis of implementation effectiveness. *BMC Health Serv. Res.* 15:6 10.1186/s12913-014-0657-3I PMC4307151 25608564

[B21] Lewy H. (2015). Wearable technologies–future challenges for implementation in healthcare services. *Healthc. Technol. Lett.* 2 2–5. 26609396 10.1049/htl.2014.0104 PMC4614314

[B22] Livsglede for eldre (2016). *Livsglede for Eldre.* Available online at: https://livsgledeforeldre.no/livsglede-for-eldre-engelsk/ (accessed January 18, 2021).

[B23] Nøst T. H. Frigstad S. A. André B. (2016). Impact of an educational intervention on nursing diagnoses in free-text format in electronic health records. *Nord. J. Nurs. Res.* 37 100–108.

[B24] Richards D. Hallberg I. R. (2015). *Complex Interventions in Health. An Overview of Research Methods.* New York: Routledge.

[B25] Rinnan E. André B. Drageset J. Garåsen H. Espnes G. A. Haugan G. (2018). Joy of life in nursing homes: a qualitative study of what constitutes the essence of Joy of life in elderly individuals living in Norwegian nursing homes. *J. Scand. J. Caring Sci.* 32 1468–1476. 10.1111/scs.12598 30070384

[B26] Schultz J. S. André B. Sjøvold E. (2016). Managing innovation in eldercare: a glimpse into what and how public organizations are planning to deliver healthcare services for their future elderly. *Int. J. Healthc. Manag.* 9, 1–12.

[B27] Schultz J. S. Sjøvold E. André B. (2017a). Can formal innovation training improve group-and organizational-level innovativeness in a healthcare setting? *J. Innov. Entrep.* 6:13.

[B28] Schultz J. S. Sjøvold E. André B. (2017b). Can work climate explain innovative readiness for change? *J. Organ. Change Manag.* 30 1–12. 10.1108/JOCM-06-2016-0112

[B29] Sjøvold E. (2004). The complexity of group development. *Int. J. Psychol.* 39 227–227.

[B30] Sjøvold E. (2006). Maturity and effectiveness in small groups. *Nord. Psychol.* 58 43–56. 10.1027/1901-2276.58.1.43

[B31] Sjøvold E. (2007). Systematizing person-group relations (SPGR) – a field theory of social. *Small Group Res.* 38 615–635. 10.1177/1046496407304334

[B32] Statistics Norway SSB (2020). *Care Services.* Available online at: https://www.ssb.no/helse/statistikker/pleie

[B33] Stone P. Harrison M. I. Feldman P. Linzer M. Peng T. Roblin D. (2005). Organizational climate of staff working conditions and safety—an integrative model. *Adv. Patient Safety* 2 467–481. 21249823

[B34] Strobe W. (2008). *Social Psychology and Health*, 3 Edn. Philadelphia: Open University Press.

[B35] Uğur S. Acuner A. M. Göktaş B. Şenoğlu B. (2007). Effects of physical environment on the stress levels of hemodialysis nurses in Ankara Turkey. *J. Med. Syst.* 31 283–287. 17685152 10.1007/s10916-007-9066-z

[B36] Weiner B. J. (2009). A theory of organizational readiness for change. *Implement. Sci.* 4:67. 10.1186/1748-5908-4-67 PMC2770024 19840381

[B37] WHO (2018). *World Ageing and Health*: *Retrieved from Helse- og omsorgsdepartementet (2012–2013).* Morgendagens omsorg. (nr 29). Geneva: WHO

